# Impact of thermal soil treatment on heavy metal mobility in the context of waste management

**DOI:** 10.1177/0734242X241251398

**Published:** 2024-05-09

**Authors:** Daniel Vollprecht, Theresa Sattler, Julia Kern, Iris Berrer, Roland Pomberger

**Affiliations:** 1 Montanuniversitaet Leoben, Chair of Waste Processing Technology and Waste Management, Leoben, Austria; 2 University of Augsburg, Institute of Materials Resource Management, Chair of Resource and Chemical Engineering, Augsburg, Germany; 3 VTU Engineering GmbH, Vienna, Austria

**Keywords:** Heavy metals, contaminated soils, thermal treatment, leaching, mineralogy, hydrogeochemical modelling

## Abstract

Thermal soil treatment is a well-established remediation method to remove organic contaminants from soils in waste management. The co-contamination with heavy metals raises the question if thermal soil treatment affects heavy metal mobility in soils. In this study, four contaminated soils and a reference sample were subjected to thermal treatment at 105°C, 300°C and 500°C for 7 day. Thermogravimetry and differential scanning calorimetry were used to understand the reactions, and resulting gases were identified by Fourier-transformed infrared spectroscopy. Treated and untreated samples were characterised by X-ray diffraction (XRD) and electron microprobe analysis and subjected to pH-dependent leaching tests, untreated samples additionally by X-ray-fluorescence (XRF) and inductively coupled plasma mass spectroscopy (ICP-MS). Leachates were analysed using ICP-MS and ion chromatography. Maximum available concentrations were used for hydrogeochemical modelling using LeachXS/Orchestra to predict leaching control mechanisms. Leaching experiments show that thermal treatment tends to decrease the mobility at alkaline pH of Pb, Zn, Cd, As and Cu, but to increase the mobility of Cr. In the acidic to neutral pH range, no clear trend is visible. Hydrogeochemical modelling suggests that adsorption processes play a key role in controlling leaching. It is suggested that the formation of minerals with a more negatively charged surface during thermal treatment are one reason why cations such as Pb^2+^, Zn^2+^, Cd^2+^ and Cu^2+^ are less mobile after treatment. Future research should focus on a more comprehensive mineralogical investigation of a larger number of samples, using higher resolution techniques such as nanoscale secondary ion mass spectrometry to identify surface phases formed during thermal treatment and/or leaching.

## Introduction

Thermal treatment of soils is used to evaporate or decompose organic contaminants. Excavated organically contaminated soils represent a large stream of partially hazardous wastes. Prior to excavation, contaminated soils do not represent a waste and fall under another legal regime, but still threatening groundwater and the biosphere. In situ thermal desorption (ISTD) is an approach in which contaminated soil is heated to vapourise chlorinated volatile organic compounds such as trichloroethylene, which are then extracted with the soil vapour (Heron et al., 2009). The removed contaminants are adsorbed on activated carbon or destroyed by catalytic post-combustion (Dörrie & Längert-Mühlegger, 2010). In the ISTD, the saturated zone is heated to 100°C and the unsaturated zone even to 250–300°C to mobilise adsorbed and dissolved contaminants by increased water solubility and vapour pressure, co-distillation, reduced viscosity and density and accelerated desorption of organic contaminants (Heron et al., 2009). Recent developments in a specific variant of ISTD, thermal conductive heating, indicate the potential of this method for broader applications (Bittens & Beutle, 2022; Schönberg et al., 2022; Vollprecht et al., 2022).

The impact of ISTD on metal mobility is rarely investigated. Roh et al. (2000) showed an increase in the mobility of Fe and Al. Bonnard et al. (2010) explained a higher ecotoxicity by a change in heavy metal speciation. Biache et al. (2008) observed not only a weaker sorption due to destruction of organic matter but also a redistribution of Fe and Zn into less soluble fractions. Wang et al. (2016) found for soils from an e-waste processing site that off-site thermal treatment at 700°C reduced the leaching of Be, Cr, Co, Ni, Cu Zn and Cd. However, at these temperatures the total concentrations of some heavy metals such as Pb and Cd in the soil decrease due to evaporation (Chou et al., 2010). Huang et al. (2011) found that thermal treatment at 550°C redistributes Cr, Cu, Ni and Pb from Fe- and Mn-bound forms to acid-extractable, organic-matter-bound and residual forms. This means that a moderately mobile fraction is redistributed into more and less soluble fractions, without a clear trend regarding overall leaching. Dong et al. (2014) demonstrated that the destruction of organic matter at 400–500°C leads to a decreased sorption of Cu and Cd, but to a higher sorption of Pb, possibly due to the interaction with newly formed aromatic substances.

Consequently, the existing studies on the effect of thermal soil treatment on (heavy) metal mobility are scarce and contradictory. The few studies on this topic are based on the purely empirical principle of sequential extraction, which does neither consider the real mechanisms controlling mobility nor investigate the real speciation of heavy metals (Vollprecht et al., 2020). Therefore, the aim of this study is to determine the effect of thermal treatment on the heavy metal mobility in soils to investigate whether additional care needs to be taken in ISTD projects to prevent unintended heavy metal mobilisation or whether ISTD could have the positive side effect of immobilising heavy metals when applied to co-contaminated soils. Consequently, the findings of this study will allow a more sustainable treatment of the huge waste flow of soils contaminated with both organic and inorganic pollutants.

## Materials and methods

Four samples of contaminated soils and one uncontaminated reference sample were selected. The selection criteria were the presence of both organic pollutants, which could be thermally treated, and heavy metals, whose mobility would be investigated as a function of prior thermal soil treatment. However, due to the lack of suitability of several sites, only two sites with both inorganic and organic contaminations, and two sites with only inorganic contaminations could be sampled. The reference sample was obtained from a composting plant and serves as a non-contaminated reference material. An overview of the samples used in this study is given in Table 1.

**Table 1.** Samples used within this study.

Sample name	Organic contaminants	Inorganic contaminants
ST30	Hydrocarbons	Cr
ST32	None	Pb, Zn
N82	Mineral oil hydrocarbons	Pb, Zn, Cu
K29	None	Pb, As
Reference	None	None

Sampling was based on ÖNORM S2091 (Austrian Standards, 2006). Samples (>55 kg) were taken by hand with shovels at the surface. The samples were homogenised and screened at 2 mm. The screen underflow was used for further analyses, as only this fraction is relevant for surface-controlled processes such as contaminant mobility. Subsamples of approximately 600–1000 g were used for thermal treatment in containers of 820 cm³. In order to produce sufficient treated material for further experiments (3 kg), several identical experiments were carried out. The thermal treatment was conducted in a drying cabinet (Memmert 700) at 105°C in a muffle furnace (Heraeus) at 300°C and in another muffle furnace (Nabertherm S27) at 500°C for 7 day each. These temperatures were selected as they cover the range used in ISTD. Untreated and treated samples were characterised for chemical and mineralogical composition as well as for heavy metal mobility. To determine the organic contaminants in the original samples, the hydrocarbon index was determined according to EN 14039 (DIN Deutsches Institut für Normung e.V., 2005) and the polycyclic aromatic hydrocarbons (sum of 16/EPA as well as benzo(a)pyrene) according to ÖNORM L1200 (Austrian Standards, 2003) at ESW Consulting Wruss ZT GmbH. The bulk chemical composition of the original samples was determined using a pressed tablet at CRB GmbH according to CRB PA03:1013-09. The loss on ignition (LOI) was determined according to DIN EN ISO 26845 at 1025°C (DIN Deutsches Institut für Normung e.V., 2008). For the determination of trace elements, the original samples were digested. Total digestion according to ÖNORM EN 13656 (Austrian Standards, 2021) was used to prepare the samples for a semi-quantitative screening using inductively coupled plasma mass spectroscopy (ICP-MS, Agilent 7500ce). Calorimetric digestion according to ÖNROM EN 14582 (Austrian Standards, 2016) was conducted to prepare the samples for ion chromatography (IC, DIONEX ICS-2000) to determine semi-quantitatively the contents of chlorine and fluorine. Alkali digestion of those original samples containing chromium according to former investigations was conducted to prepare the sample for Cr(VI) determination using 1.5-diphenylcarbazide according to DIN 38405-24 (DIN Deutsches Institut für Normung e.V., 1987). All chemical analyses were conducted at the Chair of Waste Processing Technology and Waste Management, Montanuniversitaet Leoben. In order to predict and understand the behaviour of the samples during thermal treatment, a combination of differential scanning calorimetry (DSC) and thermogravimetry (TG) was applied using a simultaneous thermal analyser (Netzsch) at the Chair of Process Engineering of Industrial Environmental Protection, Montanuniversitaet Leoben. About 65 mg of material was used, and the samples were heated at 10°C minute^−1^ up to 1000°C in both air and argon. The off-gas was analysed for by Fourier-transformed infrared spectroscopy to detect molecules such as H_2_O and CO_2_. Untreated samples and those treated in the muffle furnace were subjected to pH-dependent leaching tests according to EN 14997 (DIN Deutsches Institut für Normung e.V., 2015) for 8 pH values per sample using NaOH or HNO_3_ for pH adjustment (liquid:solid ratio 1:10, *t* = 48 h). Leachates were analysed by ICP-MS (Agilent 7500ce) for Li, Be, Na, Al, Mg, Si, P, K, Ca, Ti, V, Cr, Mn, Fe, Co, Ni, Cu, Zn, As, Se, Sr, Mo, Pd, Ag, Cd, Zn, Sb, Te, Ba, W, Tl and Pb, and by IC (DIONEX ICS-2000) for fluoride, chloride, sulphate and phosphate. Untreated and treated samples were analysed for phase composition by X-ray diffraction (Panalytical X’Pert Pro, Co Kα radiation (1.78901 Å) step size 0.008° 2 Ө, measurement time per step 40 s, sample rotation) at the Institute of Applied Geosciences at Graz University of Technology. Reflected light microscopy (Olympus with EOS Utility Program) of treated and untreated samples was conducted at the Chair of Resource Mineralogy, Montanuniversitaet Leoben, to identify highly reflective areas as candidates for heavy metal bearing mineral phases. Electron microprobe analysis (EMPA, JEOL Superprobe JXA 8200, Freising, Germany) was applied for untreated and treated samples at the Eugen F. Stumpfl laboratory at the Chair of Resource Mineralogy, Montanuniversitaet Leoben. Samples were coated with graphite using a sputter coater (EmiTEch K950X). Backscattered Electron (BSE) images were used to identify possible heavy metal phases, taking in account prior information from reflected light microscopy. In these areas (200 × 200 µm), elemental mappings (30 nA, 156 kV) of Al, S, Mg, K, Ti, O, P, Fe, Ca, Si and Pb, and for those samples containing significant amounts of C, Zn, Mn, Ba, As, Cl, Cr, Na and Zr according to XRF also of those elements, were conducted. Hydrogeochemical modelling (LeachXS™/Orchestra) was used to reveal the leaching controlling mechanisms following in the principle the modelling approach of Neuhold et al. (2019). Maximum concentrations from pH-dependent leaching tests were used as ‘available concentrations’ for modelling. As the amount of reactive hydrous ferrous oxide (HFO) and the concentration of CO_3_^2−^ in the leachate are not known, these parameters were fitted to the measured leaching data. Calculations were based on the minteqv4.dat database (USGS, Reston, Virginia, USA). The sum of pH and pE was set to 10. As a first step, all mineral phases present in the database were considered. Then, subsequently, mineral phases, whose precipitation underestimated the experimentally obtained leachable concentrations, were removed from the model. Finally, the heavy metal containing mineral phases determined via EMPA, and the leaching controlling mechanisms determined via LeachXS™ (Vanderbilt University, Nashville, Tennessee, USA), were compared to validate the hypothesis derived from modelling.

## Results and discussion

### Sample characterisation

Chemical analyses of the investigated samples confirmed that sample N82 is heavily contaminated with organic pollutants (3600 mg kg^−1^ hydrocarbons, 138 mg kg^−1^ PAH, 9 mg kg^−1^ benzo(a)pyrene), sample ST30 is characterised by a hydrocarbon index of 200 mg kg^−1^, which is equal to the inspection value for contaminated sites in Austria, whereas the other three samples do not contain relevant organic contaminations. XRF analyses (Table 2) show that the samples ST30 and the Reference sample are characterised by high SiO_2_ and lower CaO concentrations, whereas the ST32, N82 and K29 have comparatively lower SiO_2_, but higher CaO concentrations, suggesting that the former two samples are richer in silicates, whereas the latter two contain significant amounts of carbonates. Regarding heavy metals, the presence of Cr in ST30, Pb and Zn in ST32, Pb, Zn and Cu in N82 and Pb in K29 was proven, whereas the reference samples did not show increased concentrations.

**Table 2.** Chemical composition of the investigated samples according to XRF in wt% (only main elements >1 wt% and heavy metals above the detection limit are displayed, bold values mark increased heavy metal contents).

Chemical component	ST30	ST32	N82	K29	Reference
Na_2_O	1.00	0.27	0.20	0.90	1.97
MgO	3.05	1.82	4.85	6.18	4.47
Al_2_O_3_	11.14	11.74	7.76	7.57	14.19
SiO_2_	51.51	38.19	28.82	38.87	59.37
P_2_O_5_	0.57	1.71	0.34	0.21	0.48
K_2_O	1.75	2.85	0.95	1.21	2.22
CaO	5.53	8.75	12.20	18.72	2.69
TiO_2_	0.81	1.87	0.34	0.43	0.62
Cr_2_O_3_	**0.05**	<0.03	<0.03	<0.03	<0.03
Fe_2_O_3_	5.88	12.63	7.15	3.76	5.62
CuO	<0.03	**0.05**	**1.15**	<0.03	<0.03
ZnO	**0.05**	**0.59**	**0.28**	<0.03	<0.03
BaO	0.03	1.02	0.08	<0.03	0.05
PbO	<0.03	**1.32**	**0.48**	**0.04**	<0.03
LOI	17.91	15.91	34.44	21.67	7.86

Total digestion followed by ICP-MS yielded further information on heavy metal concentrations in the investigated soil samples. Besides the above-mentioned elements also Ni, As and Cd were detected, the two latter partly exceeding the inspection values for contaminated sites in Austria (Austrian Standards, 2018), whereas the reference sample was below these values (Table 3).

**Table 3.** Heavy metal contents (mg kg^−1^ DM) determined by ICP-MS after total digestion, compared with the inspection values for contaminated sites according to ÖNORM S 2088-1.

Chemical element	ST30	ST32	N82	K29	Reference	Limit values
Cr	320	82	64	39	90	100/500
Ni	60	45	84	22	93	100/500
Cu	51	310	8880	26	28	100/5000
Zn	350	3370	2470	73	100	500/1500
As	10	<25	100	33	<25	50/200
Cd	0.46	13	9.0	<2.5	<2.5	2/10
Pb	50	12,600	5420	310	34	100/500

Calorimetric digestion turned out to be incomplete, so Cl and F concentrations obtained by IC from the digestates lying in the range of 220–2320 mg kg^−1^ Cl and up to 250 mg kg^−1^ F represent not the actual concentrations. Alkaline digestion of sample ST30 followed by photometric determination of Cr(VI) yielded a value of 2.36 mg kg^−1^ DM indicating that less than 1% of the total Cr is present as Cr(VI).

**Figure 1.**
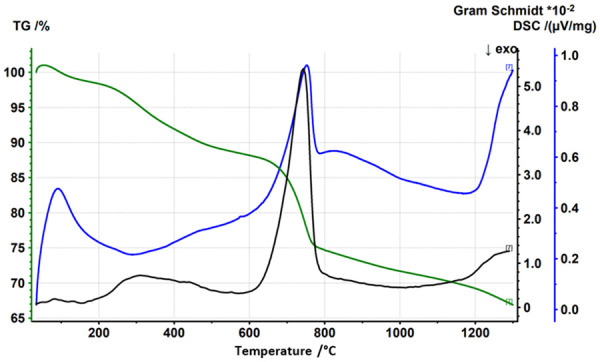
DSC, TG and Gram Schmidt plot of sample N82 under air.

DSC/TG analyses of all five samples under air showed similar results. The greatest mass loss was for sample N82 (37%), which is consistent with the highest LOI for this sample. The reference sample showed the lowest mass loss (8%) which is also consistent with the LOI, and all other samples reveal mass losses of about 20%. In the range of 30 to 200°C an endothermic first mass loss of 1–5% is observed which is mainly CO_2_ according to the Gram Schmidt plot, indicating the decomposition of organic matter. Between 200 and 400°C, an exothermic loss of mass was observed (7–12% for samples ST30, ST32, N82; 2% for samples K29 and reference), representing the combustion of organic matter. Between 600 and 800°C, a third mass loss occurs, which can be explained by the calcination of carbonates and is associated with the release of CO_2_ according to the Gram Schmidt plot and an endothermic reaction. Under reducing conditions the TG curves are very similar to those in air. Interestingly, despite the absence of oxygen, the Gram Schmidt plot again shows the release of CO_2_, which means that the samples themselves contained sufficient oxygen to allow this reaction. An example of the results of thermoanalysis is given in Figure 1.

As suggested by TG, thermal treatment of the samples in the muffle furnace resulted in significant mass losses. Samples with organic contaminations (ST30, N82) lost about 50% of their weight at 500°C, whereas the other samples lost only 20–30%. This can be explained by the high organic content of the two former soils. All samples turned from greyish to reddish when treated at 300°C or above, which can be explained by the oxidation of Fe^2+^ to Fe^3+^.

Reflected light microscopy indicates that treated samples appear much brighter due to the decomposition of organics. Furthermore, more and more fractures are seen as the treatment temperature increases.

The XRD patterns of samples K29 and N82 show that already after a treatment at 500°C portlandite, Ca(OH)_2_, occurs, which must have formed during cooling by the reaction of free lime, CaO, with atmospheric moisture. Additionally, in sample N82 the share of hematite, Fe_2_O_3_, increases to the expense of magnetite, Fe_3_O_4_, indicating an oxidation of Fe(II) to Fe(III). XRD patterns of sample ST30 and ST32 and the reference sample show an increasing share of chlorite, (Mg, Fe^2+^, Al)_3_[(OH)_2_|AlSi_3_O_10_] • (Mg, Fe^2+^, Al)_3_(OH)_6_, and muscovite, KAl₂[(OH, F)₂|AlSi₃O₁₀], with increasing treatment temperature and a decomposition of kaolinite, Al₄[(OH)₈|Si₄O₁₀], at 500°C.

### pH-dependent leaching, hydrogeochemical modelling and electron microprobe analysis

**Figure 2.**
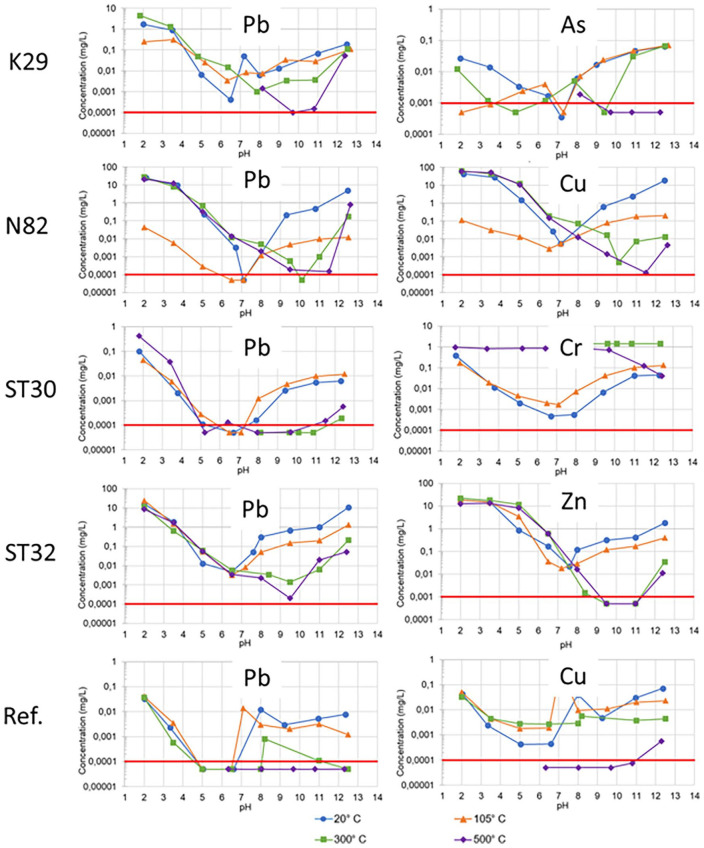
pH-dependent leaching of selected heavy metals from untreated samples and samples treated for 7 day at the given temperatures. Red line = limit of detection. The extended measuring uncertainty is about 30% of the measured concentration which is in the range of the size of the symbols used as data spots. Data are displayed logarithmically.

**Figure 3.**
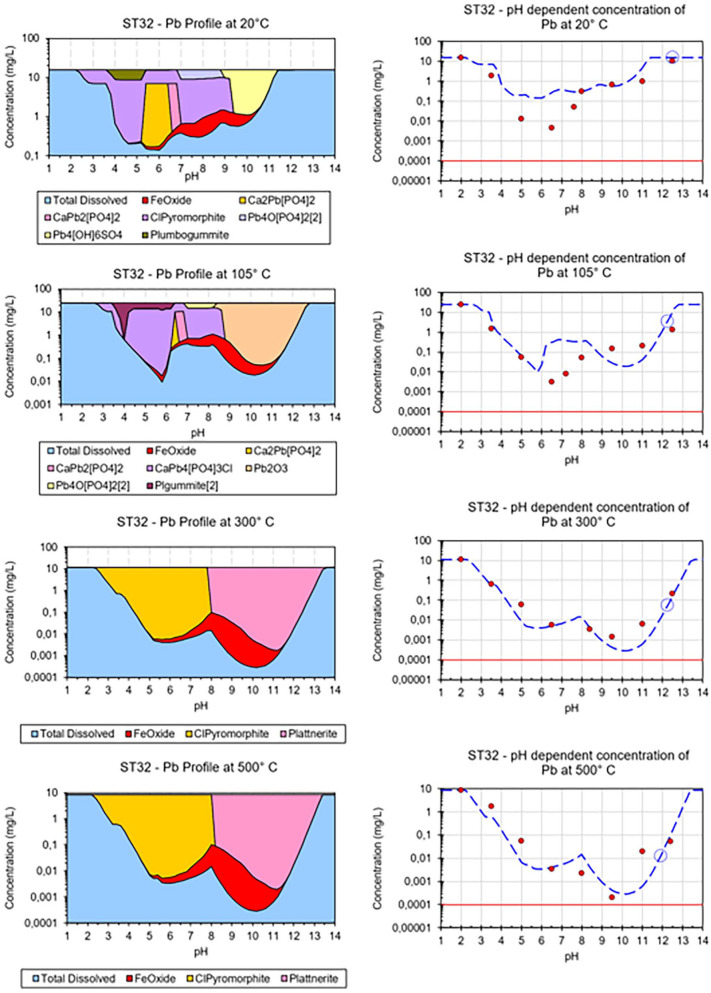
Left: Hydrogeochemical modelling of the pH-dependent leaching of Pb from the untreated and thermally treated sample ST32. Right: Comparison of measured data (red circles) and modelled data (dashed blue lines). Red line = limit of detection.

**Figure 4.**
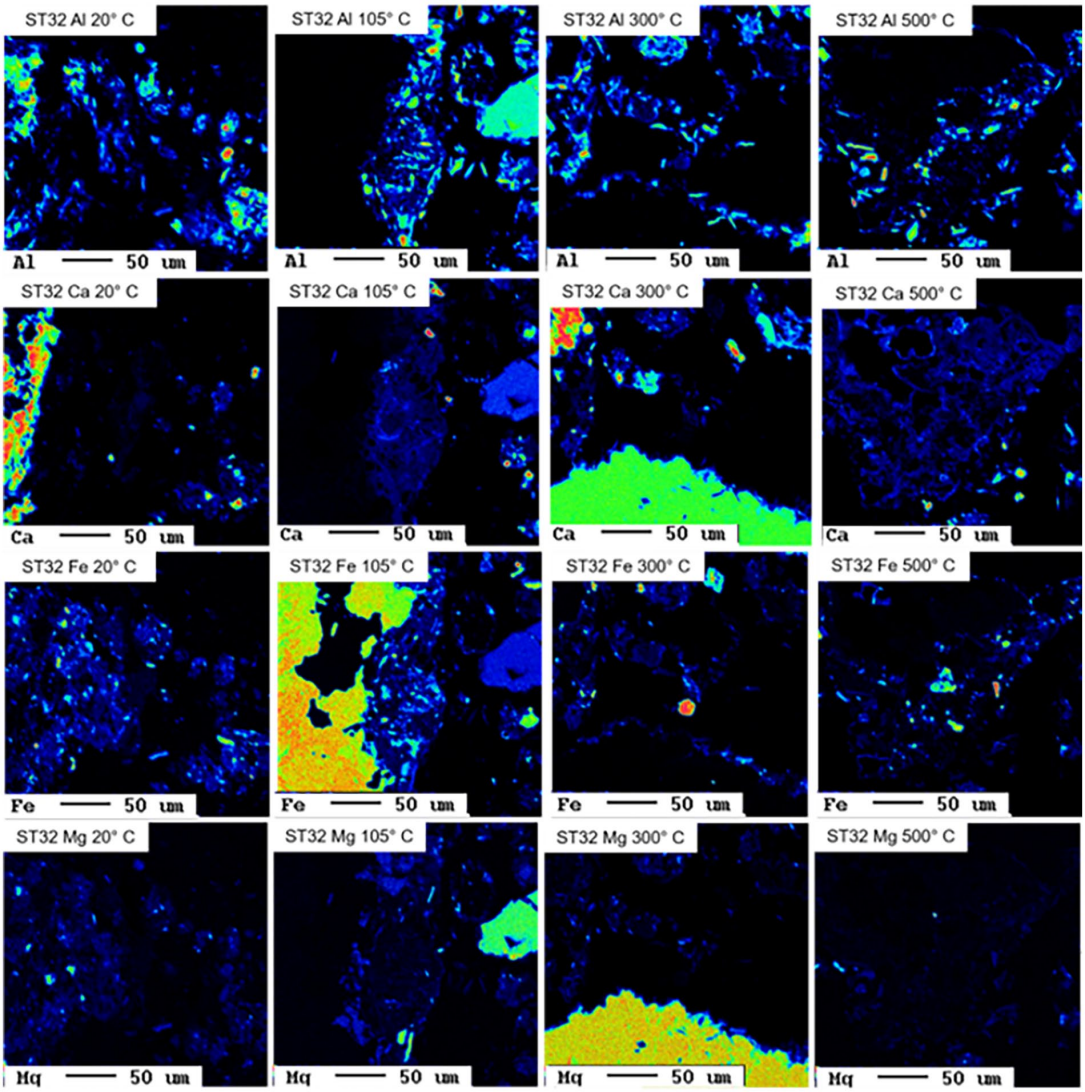
Elemental distribution maps Al, Ca, Fe and Mg in the untreated and thermally treated sample ST32.

**Figure 5.**
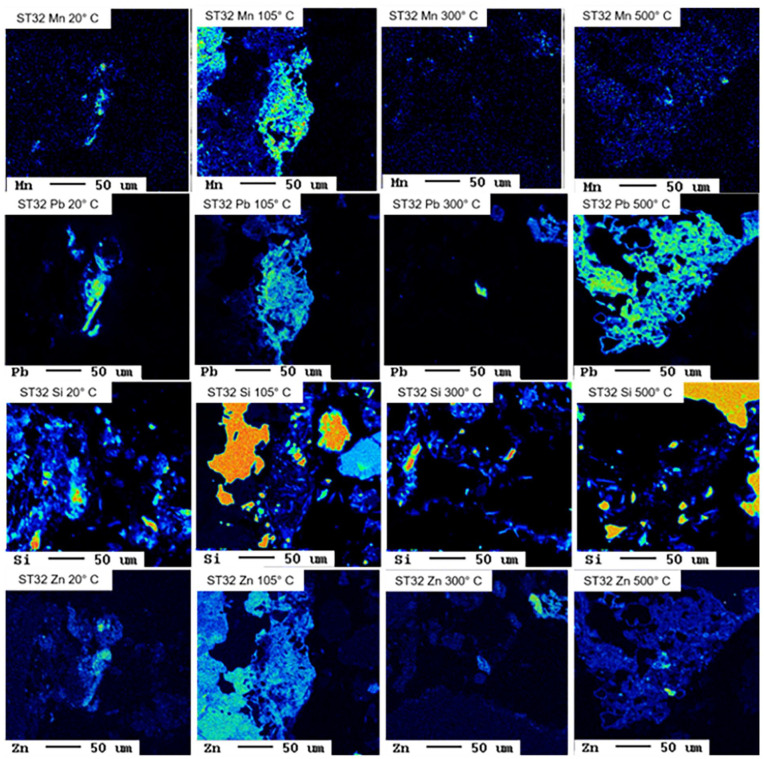
Elemental distribution maps Mn, Pb, Si and Zn in the untreated and thermally treated sample ST32.

Selected results from pH-dependent leaching tests are displayed in Figure 2, selected hydrogeochemical models in Figure 3, and selected EMPA results in Figures 4 and 5.

pH-depending leaching tests of sample K29 showed that for Pb the maximum leaching is in the acidic pH range, whereas for As even higher leaching is observed at alkaline pH. This can be explained by the cationic character of Pb^2+^ versus the anionic character of AsO_4_^3−^. Thermal treatment of the soil at 300°C and even more at 500°C decreased the leaching of Pb in the alkaline range. A similar trend was observed for As. However, since the charge of the dissolved species of these two elements is different, other processes, for example the stronger adsorption of Pb to more negatively charged surfaces of newly formed minerals versus the precipitation of secondary arsenate phases with cations released from thermally unstable minerals, might be an explanation. In contrast, there is no clear trend visible in the acidic pH range, suggesting kinetic effects in the leaching experiment as an explanation.

Hydrogeochemical modelling of the pH-dependent leaching of sample K29 suggests that As leaching is controlled by adsorption onto HFO phases, and correctly predicts decreased leaching with increasing treatment temperature in the alkaline pH range, but overestimates the experimentally observed leaching in the acidic pH range. Pb release is proposed to be controlled by the solubility equilibrium of plattnerite (β-PbO_2_) in the alkaline pH range, and of pyromorphite (Pb_5_[Cl|(PO_4_)_3_]) in the acidic pH range, as well as by the adsorption of initially released Pb^2+^ onto HFO phases. The modelling results describe well the observed leaching maximum in the neutral to slightly alkaline pH range and also the trend of decreasing leaching after thermal treatment due to the decreased solubility of plattnerite. EMPAs of sample K29 showed the association of Pb with P and partially Ca and Cl, independent from treatment temperature, suggesting the presence of phosphohedyphan (Ca_2_Pb_3_(PO_4_)_3_Cl), whereas the mineralogical bonding of As could not be revealed. Thus, the mineral phases present according to analytical methods and the ones predicted by hydrogeochemical modelling are not the same. This can be explained by the conceptual model that the bulk phases present are not the ones controlling the leaching, that is they are not the ones with the lowest solubility, but secondary mineral phases may form at their surfaces, invisible for the EMPA, but relevant as leaching-decreasing factors.

pH-dependent leaching of sample N82 shows a minimum leaching of Cu at pH 7 for the untreated sample and the sample treated at 105°C, whereas thermal treatment at 300°C or above leads to a steady decrease in leaching with increasing pH. For Pb, a similar trend as for sample K29 is observed. At alkaline pH, the leaching decreases with increasing treatment temperature. The same trend is observed for Zn. Decreasing leaching of Cu, Pb and Zn in the alkaline pH with increasing temperature is suggested to be due to the same mechanisms as described for Pb in sample K29. In contrast, in the acidic pH range, there is no clear trend visible, which agrees with sample K29.

Hydrogeochemical modelling of pH-dependent leaching of sample N82 suggests that leachates are in sorption equilibrium with HFO phases for the untreated sample and the sample treated at 105°C, whereas leachates of samples treated at 300°C and 500°C are in dissolution equilibrium with spertiniite, Cu(OH)_2_ and tenorite, CuO, respectively. This suggests that thermal treatment leads to relocation of Cu into low soluble mineral phases. For Pb, modelling results also indicate a decreasing leaching at alkaline pH after thermal treatment which is explained by the model by the low solubility of plattnerite (β-PbO_2_). In contrast, the decrease in Zn leaching at alkaline pH is not well predicted by the model. With increasing treatment temperature, the measured leachable concentrations deviate more and more from the calculated concentrations in equilibrium with zincite (ZnO), Ca zincate (CaZn_2_(OH)_6_·2H_2_O) and adsorption onto HFO. This suggests either the presence of low-soluble phases not present in the database or the kinetically suppressed dissolution of higher soluble mineral phases. EMPA results reveal the presence of Cu, Pb and Zn in different mineral phases of complex compositions. Cu is not only finely disseminated in the samples but also shows enrichments in CuO or Cu(OH)_2_ in thermally treated samples, supporting the hypothesis obtained from hydrogeochemical modelling. Pb occurs together not only with Al, Si and O but also in other phases, and no clear trend with treatment temperature can be seen. Zn is finely disseminated in the sample, with a slight tendency to be associated with silicates in the sample treated at 500°C. In summary, there is some information supporting the idea of decreased leaching of Cu, Pb and Zn due to the formation of Pb- and Cu-oxides and Zn-silicates during thermal treatment.

pH-dependent leaching of sample ST30 shows a similar temperature dependence of the leaching of Cu, Pb and Zn as in the other samples, that is a decrease in leaching in the alkaline pH range due to thermal treatment and no effect in the acidic range. In contrast, for Cr, which is the most relevant contaminant in this sample, the leaching increases by thermal treatment, especially in the neutral range. Hydrogeochemical modelling suggests that the very low leaching of Cr in the neutral pH range in the untreated sample is due to strong sorption to HFO phases. The increased mobility after thermal treatment is not reflected in the hydrogeochemical model. However, it seems reasonable that the thermal treatment dehydroxylates HFO phases creating iron oxides with much lower specific surface area and accordingly lower sorption capacity for Cr. It has to be considered that the equilibrium between Cr(III) and Cr(VI) is shifted towards Cr(VI) with increasing temperature. Thus, it is suggested that Cr is oxidised during thermal treatment, and the formed Cr(VI) species are more mobile than Cr(III) in untreated soils. This is in agreement with Cr(VI) measurements indicating increasing leachable Cr(VI) concentrations from <0.5 mg kg^−1^ DM in the untreated sample via 2.0 and 5.2 mg kg^−1^ DM for samples treated at 105°C and 300°C to 6.0 mg kg^−1^ DM for the sample treated at 500 mg kg^−1^ DM. EMPA results suggest the presence of Cr as chromite (FeCr_2_O_4_) in the untreated sample, whereas in the treated samples no Cr phase was identified. However, the thermal stability of chromite is much higher, so this observation is only due to the nugget effect. The suggested oxidation of Cr(III) to Cr(VI) is thought to take place only at the surface and let the chromite grains intact.

pH-dependent leaching of sample ST32 reveal decreasing leaching in the alkaline pH of Cd, Zn and Pb with increasing treatment temperature, which is in line with observations for Pb and Zn and other samples and demonstrates the geochemical similarity between Pb and Cd. Hydrogeochemical modelling suggests sorption to HFO phases as leaching controlling mechanism for Cd, but fails to explain the temperature effect, since the untreated sample and the sample treated at 105°C show higher leaching in the alkaline pH range than suggested by the model. Interestingly, for samples treated at 300 and 500°C modelled and measured leachable concentrations of Cd match very well. This suggests that the sorbents are produced during thermal treatment, but further research is required to find explanations. Adsorption on HFO phases is also suggested to be the leaching controlling mechanism for Zn up to pH 12, but the corresponding model overestimates the leaching between pH 9 and 12 significantly. The decrease in Pb leaching in the alkaline range due to thermal treatment is reflected well in the hydrogeochemical models. As for the other Pb-containing samples, the formation of plattnerite (β-PbO_2_) during thermal treatment plays a key role here. Interestingly, for the untreated sample, the model suggests leaching control by Pb_4_(OH)_6_SO_4_, a Pb(II) phase, for the sample treated at 105°C, Pb_2_O_3_, a Pb(III) phase, and for samples treated at higher temperatures Pb(IV)-containing plattnerite are suggested. Thus, oxidation of Pb during thermal treatment might be an explanation of decreased leaching in subsequent experiments. However, the EMPA results indicate an association of Pb and Zn with Mn in the untreated sample as well as in the sample treated at 105°C, with Fe, S and O in the sample treated at 300°C, and with Ca, Mn and P can be seen in the sample treated at 500°C. The lacking consistency between modelling results and EMPAs suggests that certain leaching-controlling phases are present in small amounts at the interface, whereas the variety of bulk phases are not the ones controlling the leaching of Pb and Zn.

Not only did the reference sample contain much lower amounts of heavy metals, but also lower leaching of these metals. However, pH-dependent leaching tests confirmed the trend of decreasing leaching of Pb, Cu and Zn. Hydrogeochemical modelling suggests adsorption to HFO as leaching controlling mechanism for all three metals, but fails to predict the suppressive effect of thermal treatment on leaching. EMPAs revealed the bonding of Pb, Cu and Zn to iron sulphate in the untreated sample and the disperse distribution of these elements in treated samples.

In the context of waste management, these findings contribute to waste minimisation. Contaminated soil prior to excavation is not a waste, and in situ treatment is a sustainable alternative to excavation, that is waste generation, and ex situ waste treatment. If the samples investigated within this study had been excavated, they would have to be landfilled at a residual waste landfill, the highest landfill class in Austria for non-hazardous wastes (Austria, 2008). In other cases, excavated contaminated soils even represent hazardous waste, highlighting the relevance of our results also for hazardous waste management.

## Conclusion

Four heavy metal contaminated soils and one reference soil were characterised for chemical and mineralogical composition. The samples were treated for 7 day at temperatures of 105, 300 and 500°C. Untreated and treated samples were subjected to pH-dependent leaching tests. Leaching experiments show that thermal treatment tends to decrease the mobility at alkaline pH of Pb, Zn, Cd, As and Cu but increase the mobility of Cr. In the acidic to neutral pH range, no clear trend is visible. Hydrogeochemical modelling suggests that adsorption processes play a key role as leaching controlling mechanisms. It is suggested that formation of minerals with a more negatively charged surface during thermal treatment are one reason why cations such as Pb^2+^, Zn^2+^, Cd^2+^ and Cu^2+^ are less mobile after treatment. Oxidation of Pb(II) to Pb(IV) during thermal treatment is suggested to decrease subsequent leaching, whereas oxidation of Cr(III) to Cr(VI) has the inverse effect. For Pb and Cu, the formation of low soluble oxide phases such as tenorite and plattnerite, during thermal treatment is suggested as an additional immobilisation mechanism. Future research should focus on a more comprehensive mineralogical investigation of a larger number of samples and with higher resolution techniques, such as nano-scaled secondary ion mass spectrometry, to identify surface phases formed during thermal treatment and/or leaching experiments, which would allow to verify or falsify the hypotheses from hydrogeochemical modelling regarding leaching-controlling mechanisms. Regarding waste management practices, thermal treatment of contaminated sites could be applied for simultaneous extraction of volatile organic compounds and in situ immobilisation of Pb, Zn, Cd, Cu and As in alkaline soils. The presence of these metals in acidic or neutral soils does not have a negative effect, but soils with co-contamination of organic pollutants and Cr should not be treated thermally without a pump and treat system to capture the mobilised Cr(VI).
